# Trends in the COVID-19 Pandemic in Italy during the Summers of 2020 (before Mass Vaccination), 2021 (after Primary Mass Vaccination) and 2022 (after Booster Mass Vaccination): A Real-World Nationwide Study Based on a Population of 58.85 Million People

**DOI:** 10.3390/pathogens12121376

**Published:** 2023-11-22

**Authors:** Luca Roncati, Giulia Bartolacelli, Carlo Galeazzi, Stefania Caramaschi

**Affiliations:** 1 Department of Surgery, Medicine, Dentistry and Morphological Sciences with Interest in Transplantation, Oncology and Regenerative Medicine, University of Modena and Reggio Emilia, 41121 Modena, Italy; 2 Department of Maternal, Infant and Adult Medical and Surgical Sciences, University of Modena and Reggio Emilia, 41121 Modena, Italy

**Keywords:** COVID-19, SARS-CoV-2, SARS-CoV-2 Gamma variant, SARS-CoV-2 Delta variant, variant of concern (VOC), tropical and subtropical moist broadleaf forests (TSMF), vaccination, booster dose, lockdown, Italy

## Abstract

Like all RNA viruses, SARS-CoV-2 shows a high mutation rate, which has led to the emergence of new variants. Among them, Gamma and Delta developed at the turn of 2020–2021 in Amazonas and India, two ecoregions characterized by hot-humid weather, very similar to that of the summer season in Italy due to climate change, the first Western country to be hit hard by COVID-19 and to experience lockdown restrictions in a democratic framework of 58.85 million people. The aim of our research has been to evaluate the impact of climate on the COVID-19 pandemic in Italy during the summers of 2020 (before mass vaccination), 2021 (after primary mass vaccination) and 2022 (after booster mass vaccination), also taking into account the emergence of these two variants. Methods: During the state of national health emergency and the Draghi government, the Civil Defense Department released the aggregate data coming from the Ministry of Health, the Higher Institute of Health, the Independent Provinces and the Italian Regions daily, in order to inform about the pandemic situation in Italy. Among these data there were the number of deaths, hospitalizations in intensive care units (ICU), non-ICU patients, contagions and performed swabs. By means of a team effort, we have collected and elaborated all these data, comparing the COVID-19 pandemic in Italy during the summers of 2020 (following the nationwide lockdown), 2021 and 2022. Results: from the summer of 2020 to the summers of 2021 and 2022 all pandemic trend indicators have shown a sharp worsening in Italy. COVID-19 deaths increased by ≈298% and ≈834%, ICU hospitalizations by ≈386% and ≈310%, non-ICU hospitalizations by ≈224% and ≈600%, contagions by ≈627% and ≈6850% (i.e., ≈68.50 times), swabs by ≈354% and ≈370%, and the mean positivity rate passed from ≈1% to ≈2% and ≈20%, respectively. Conclusions: SARS-CoV-2 can be transmitted in any climate, including areas with hot and humid weather, and the emergence of variants adapted to hot-humid climates may result in summer COVID-19 outbreaks, even in neither tropical nor subtropical countries. Although COVID-19 vaccines can confer cross-protection against newly emerging variants, this cross-immunity is naturally not absolute but limited, considering that vaccine protection wanes significantly after 6 months. It follows that a subject vaccinated at the beginning of the winter will not be completely covered in the height of the summer, and we should not forget the unvaccinated. As a final remark, the long and strict nationwide lockdown made it possible to flatten SARS-CoV-2 circulation and, therefore, its negative impact on Italy during the summer of 2020.

## 1. Introduction

First identified at the end of 2019 in the city of Wuhan (Hubei, China), the severe acute respiratory syndrome coronavirus 2 (SARS-CoV-2) is a positive-sense single-stranded ribonucleic acid (RNA) virus, responsible for the ongoing coronavirus disease 2019 (COVID-19) pandemic. Like all RNA viruses, it shows a high mutation rate compared to deoxyribonucleic acid (DNA) viruses, because viral RNA polymerase lacks the proofreading ability of DNA polymerase [1]. This genetic instability of RNA viruses makes it difficult to produce long-lasting effective vaccines against them.

Since its discovery, many variants of SARS-CoV-2 have emerged around the world as expected. The term ‘variant of concern’ (VOC) refers to any variant of the virus where mutations in the receptor-binding domain of the spike protein substantially increase the binding affinity with the human angiotensin-converting enzyme 2 (hACE2) receptors, such as to favor its rapid spread [2]. Before being allocated to this category, an emerging variant may have been labeled a ‘variant of interest’ (VOI) or, alternately as synonyms, ‘variant under investigation’ (VUI) or else ‘variant under monitoring’ (VUM) [3].

**Figure 1:**
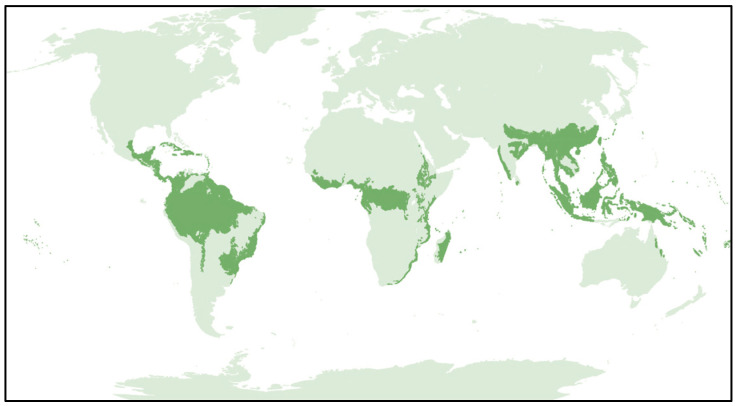
World map of tropical and subtropical moist broadleaf forests (TSMF) and ecoregions (dark green), particularly present around the Amazon basin and Congo basin, in the Caribbean and Madagascar, and in the Southeast Asia and Indian subcontinent.

To date, five VOC have been detected and designated by the World Health Organization (WHO) following the order of the Greek-letters: Alpha, Beta, Gamma, Delta and Omicron [3]. As of September 2023, only Omicron is a circulating VOC according to the aforementioned WHO [3]. Among them, Gamma and Delta developed at the turn of 2020–2021 in Amazonas and India, hence the nicknames of “Brazilian” and “Indian” variant, respectively. These two ecoregions are characterized by hot-humid weather (Figure 1), very similar to that of the summer season in Italy due to climate change, the first Western country to be hit hard by COVID-19 and to experience lockdown restrictions in a democratic framework of 58.85 million people [4].

The aim of our research has been to evaluate the trend of the COVID-19 pandemic in Italy during the summers of 2020 (before mass vaccination), 2021 (after primary mass vaccination) and 2022 (after booster mass vaccination), also taking into account the emergence of these two variants.

## 2. Materials and Methods

During the period of national health emergency and the Draghi government (dissolved on 22 October 2022), the Civil Defense Department released the aggregate data coming from the Ministry of Health, the Higher Institute of Health, the Independent Provinces (Bolzano and Trento) and the Italian Regions (Abruzzo, Basilicata, Calabria, Campania, Emilia-Romagna, Friuli-Venezia Giulia, Lazio, Liguria, Lombardia, Marche, Molise, Piemonte, Puglia, Sardegna, Sicilia, Toscana, Umbria, Valle d’Aosta, and Veneto) daily, in order to inform the population about the pandemic situation in Italy. Among these data, on a daily basis, there were the number of deaths, hospitalizations in intensive care units (ICU), non-ICU patients, contagions and performed swabs.

By means of a team effort, we have collected all these data and elaborated the respective graphs, comparing the COVID-19 pandemic in Italy during the summers of 2020 (before mass vaccination), 2021 (after primary mass vaccination) and 2022 (after booster mass vaccination).

## 3. Results

### 3.1. COVID-19 Deaths

In the summers of 2020 (20 June 2020–22 September 2020), 2021 (21 June 2021–22 September 2021) and 2022 (21 June 2022–23 September 2022) there were 1085, 3236 and 9044 deaths due to COVID-19, respectively (Table 1).

**Figure 2:**
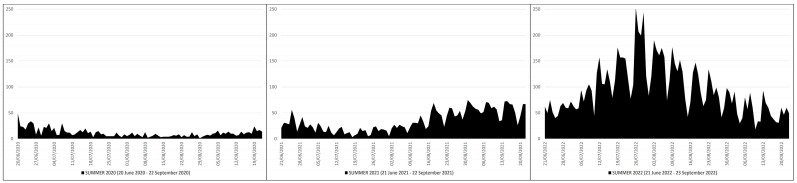
COVID-19 deaths in Italy during the summers of 2020 (20 June 2020–22 September 2020), 2021 (21 June 2021–22 September 2021) and 2022 (21 June 2022–23 September 2022) [X axis: days; Y axis: number of deaths].

This means that in the summers of 2021 and 2022 there were 2151 and 7959 more deaths than in the summer of 2020, which is equivalent to ≈2.98 and ≈8.34 times higher; in practice, from the summer of 2020 (before mass vaccination) to the summers of 2021 (after primary mass vaccination) and 2022 (after booster mass vaccination) there was an increase in deaths of ≈298% and ≈834%, respectively. The peak of deaths in the summer of 2020 (n. 49) occurred on 20 June 2020, while in the summers of 2021 (n. 75) and 2022 (n. 253) this was on 31 August 2021 and 26 July 2022 (Figure 2).

### 3.2. COVID-19 ICU Hospitalizations

**Figure 3:**
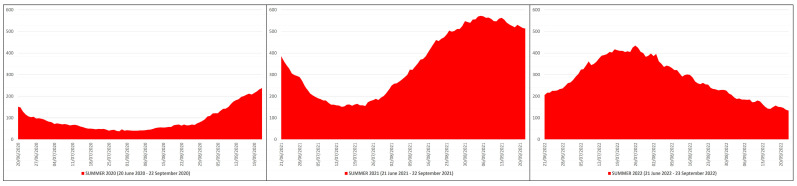
COVID-19 ICU hospitalizations in Italy during the summers of 2020 (20 June 2020–22 September 2020), 2021 (21 June 2021–22 September 2021) and 2022 (21 June 2022–23 September 2022) [X axis: days; Y axis: number of ICU hospitalizations].

In the summer of 2020, the daily ICU hospitalizations due to COVID-19 averaged 91, with a peak of 239 ICU patients on 22 September 2020, while in the summers of 2021 and 2022 the daily ICU hospitalizations averaged 351 and 282, with a peak of 572 ICU patients on 5 September 2021 and of 434 ICU patients on 26 July 2022 (Figure 3). This means that in the summers of 2021 and 2022 the daily ICU hospitalization rate was ≈3.86 and ≈3.10 times higher than in the summer of 2020; in practical terms, from the summer of 2020 (before mass vaccination) to the summers of 2021 (after primary mass vaccination) and 2022 (after booster mass vaccination) the daily ICU hospitalization rate increased by ≈386% and ≈310%, respectively.

### 3.3. COVID-19 Non-ICU Hospitalizations

**Figure 4:**
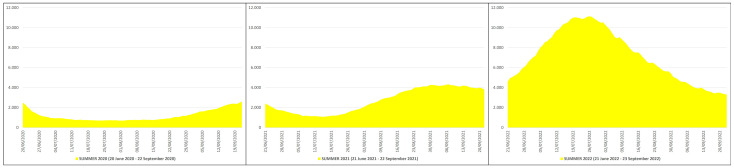
COVID-19 non-ICU hospitalizations in Italy during the summers of 2020 (20 June 2020–22 September 2020), 2021 (21 June 2021–22 September 2021) and 2022 (21 June 2022–23 September 2022) [X axis: days; Y axis: number of non-ICU hospitalizations].

In the summer of 2020, the daily non-ICU hospitalizations due to COVID-19 averaged 1198, with a peak of 2604 non-ICU patients on 22 September 2020, while in the summers of 2021 and 2022 the daily non-ICU hospitalizations averaged 2685 and 7192, with a peak of 4307 non-ICU patients on 7 September 2021 and of 11124 non-ICU patients on 26 July 2022 (Figure 4). This means that in the summers of 2021 and 2022 the daily non-ICU hospitalization rate was ≈2.24 and ≈6.00 times higher than in the summer of 2020; in practical terms, from the summer of 2020 (before mass vaccination) to the summers of 2021 (after primary mass vaccination) and 2022 (after booster mass vaccination) the daily non-ICU hospitalization increased by ≈224% and ≈600%, respectively.

### 3.4. COVID-19 Contagions

**Figure 5:**
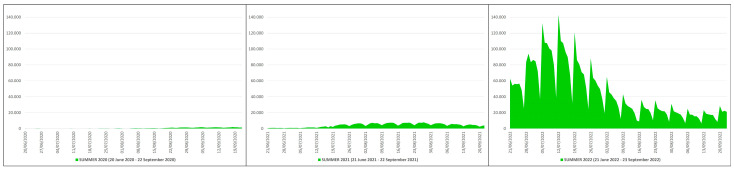
COVID-19 contagions in Italy during the summers of 2020 (20 June 2020–22 September 2020), 2021 (21 June 2021–22 September 2021) and 2022 (21 June 2022–23 September 2022) [X axis: days; Y axis: number of contagions].

In the summers of 2020, 2021 and 2022, COVID-19 contagions were 62,540, 392,322 and 4,283,835 (Table 1). This means that, from the summer of 2020 (before mass vaccination) to the summers of 2021 (after primary mass vaccination) and 2022 (after booster mass vaccination), COVID-19 contagions increased by 329,782 and 4,221,295 subjects, an increase of ≈6.27 and ≈68.50 times equal to ≈627% and ≈6850%, respectively. The peak of contagions in the summer of 2020 (n. 1907) occurred on 18 September 2020, while in the summers of 2021 (n. 7826) and 2022 (n. 142,967) on 27 August 2021 and 12 July 2022 (Figure 5).

### 3.5. COVID-19 Swab Tests

**Figure 6:**
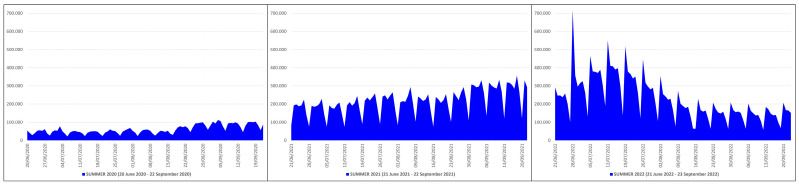
COVID-19 swab tests in Italy during the summers of 2020 (20 June 2020–22 September 2020), 2021 (21 June 2021–22 September 2021) and 2022 (21 June 2022–23 September 2022) [X axis: days; Y axis: number of swabs].

In the summers of 2020, 2021 and 2022, the number of swabs performed to detect COVID-19 were 5,717,867, 20,250,565 and 21,713,992 (Table 1). This means that, from the summer of 2020 (before mass vaccination) to the summers of 2021 (after primary mass vaccination) and 2022 (after booster mass vaccination), the swab tests increased by 14,532,698 and 15,996,125 units, an increase of ≈3.54 and ≈3.70 times equal to ≈354% and ≈370% more, respectively. The peak of performed tests in the summer of 2020 (n. 113,085) occurred on 4 September 2020, while in the summers of 2021 (n. 355,933) and 2022 (n. 717,400) on 18 September 2021 and 28 June 2022 (Figure 6).

## 4. Discussion

The spread of COVID-19 in Italy was first documented on 31 January 2020, when two Chinese tourists in Rome were found to be positive for the virus; one week later an Italian man, repatriated to Italy from Wuhan, was tested and hospitalized as the third case. From that day the situation worsened seriously, and the Italian government faced a sanitary emergency with a strict nationwide lockdown from 9 March 2020 to 3 May 2020 (included) [5].

Concretely, a ban was imposed on all citizens preventing them from leaving their homes and moving or traveling via public or private transport in a municipality other than the one in which they were located, except for proven work needs, of absolute urgency, or for health reasons. Gatherings of people in public places or places open to the public were therefore prohibited by maintaining an interpersonal distance of at least 1m in social contacts with any person not belonging to the same family unit. The access of relatives and visitors to health facilities and penitentiary or penal institutions for minors was limited; hand sanitization was recommended upon entering any place open to the public; and a mandatory quarantine was introduced for those infected and for those who had been in contact with people positive for the virus. Among the various measures adopted, there were the suspension of work activities not considered necessary for the Italian productive fabric with the exception of smart working, of educational, recreational and sporting activities (then only permitted behind closed doors), of catering services, events and initiatives of any nature, both public and private; and the closure of judicial offices, museums and other institutes or places of worship, as well as all retail commercial activities excluding those for the sale of essential goods, accessible with the use of a protective mask [5].

Thanks to a record-breaking research network, a new generation of vaccines based on the Wuhan strain had been released at the beginning of 2021 [6], and health policies had been adopted to strongly encourage free mass vaccination. To date, almost 150 million doses have been administered in Italy, where a 90.25% and an 84.89% coverage of the population over 12 has been achieved with regards to the primary vaccination cycle (two doses) and to the booster dose (three doses), respectively [7].

The undoubtful efficacy of primary or booster COVID-19 vaccination in significantly reducing deaths and ICU/non-ICU hospitalizations in adults has been well demonstrated [8,9,10,11,12]. However, from the summer of 2020 (before mass vaccination) to the summers of 2021 (after primary mass vaccination) and 2022 (after booster mass vaccination), all pandemic trend indicators have shown a sharp worsening in Italy: COVID-19 deaths increased by ≈298% and ≈834%, ICU hospitalizations by ≈386% and ≈310%, non-ICU hospitalizations by ≈224% and ≈600%, contagions by ≈627% and ≈6850% (i.e., ≈68.50 times), swabs by ≈354% and ≈370%, and the mean positivity rate passed from ≈1% to ≈2% and ≈20%, respectively.

A first explanation for these results certainly lies in the long and strict nationwide lockdown that preceded the summer of 2020 but not the summers of 2021 and 2022, allowing the circulation of SARS-CoV-2 to be flattened during the summer of 2020; in fact, thanks to the advent of specific vaccines, this drastic political measure was no longer adopted. The awareness and state of panic among the population certainly played an important role, too [13]. Conversely, despite vaccinations being made available for free and the efforts of governments, this health attention became saturated in the following summers, overwhelmed by the need to return to daily life, not dictated by the virus, with behaviors more at risk of contracting the disease. Despite this, lockdown remains the most effective measure of health policy to stop the contagions when specific or updated vaccines are not yet available, together with wearing mask indoors (preferably filtering face piece), practicing hand hygiene (with soap or hydroalcoholic solution) and keeping interpersonal social distancing (at least 1 m).

Secondly, VOC suitable for hot-humid weather, very similar to that of the summer season due to climate change in Italy, in particular around the Po basin, emerged from the summer of 2020 to the summers of 2021 and 2022, such as the Gamma and Delta variants [3]. COVID-19 virulence has been found at a maximum of <10 °C and <40 kJ/m^2^ or >60% humidity (≈70%) [14,15,16]. This behavior towards environmental temperature, solar ultraviolet radiation or relative humidity explains the reason why SARS-CoV-2 can be transmitted in any climate, including areas with hot and humid weather as stated by the WHO [17], and it is quite similar to that of other respiratory viruses, in primis influenza [18,19]. In Italy, these conditions are typical of the autumn–winter (<10 °C and <40 kJ/m^2^) or of the summer season (>60% humidity); therefore, summer outbreaks should not come as a surprise even in neither tropical nor subtropical countries, especially in the case of VOC adapted to hot-humid climate.

Thirdly, the mass vaccination campaign in Italy was implemented with vaccines based on the Wuhan strain; although vaccines developed with an antigen from a non-predominant ancestral strain can confer cross-protection against newly emerging variants of the coronavirus and vice versa [20,21], this cross-immunity is naturally not absolute but limited [22,23]. Moreover, vaccine efficacy or effectiveness against SARS-CoV-2 infection decreases from 1 month to 6 months after full vaccination by 21.0% on average (95% CI: 13.9–29.8) among people of all ages, and by 20.7% (95% CI: 10.2–36.6) among older people (at least 50 years old), and wanes considerably after 6 months [24,25]. It follows that a subject vaccinated at the beginning of the winter will not be completely covered in the height of the summer. Unfortunately, the available data do not allow us to know in what proportion the surge in the number of deaths, ICU/non-ICU hospitalizations and contagions from the summer of 2020 to the summers of 2021 and 2022 affected the 90.25% and the 84.89% of the population over 12 who were vaccinated with two or three doses, and the remaining 9.75% and 15.11%, nor do they allow us to evaluate any differences among subjects vaccinated with different types of vaccines (mRNA, subunit, viral vector). In addition, the progressive availability of rapid self-tests has surely influenced the increased number of contagions found in 2022 compared to 2020; in fact, the comparison of Figure 5 with Figure 6 shows a direct proportionality between the number of contagions and the number of swabs carried out. Elderly and frail people are more prone to have severe COVID-19 complications [26,27,28,29,30]; however, the data available on a large scale do not allow us to know the age and any comorbidities of every single patient who died, certainly a further limiting factor of this study.

## 5. Conclusions

SARS-CoV-2 can be transmitted in any climate, including areas with hot and humid weather, and the emergence of variants adapted to hot-humid climate may result in summer COVID-19 outbreaks, even in neither tropical nor subtropical countries. In the future, it is possible that similar summer outbreaks will be repeated even if numbers are unlikely to reach those of the autumn–winter season, and such as to send the healthcare system into a tailspin.

Although COVID-19 vaccines can confer cross-protection against newly emerging variants, this cross-immunity is naturally not absolute but limited, also taking into account that vaccine protection wanes considerably after 6 months. It follows that a subject vaccinated at the beginning of the winter will not be completely covered in the height of the summer, without forgetting the unvaccinated.

As a final remark, the long and strict nationwide lockdown made it possible to flatten SARS-CoV-2 circulation and, therefore, its negative impact on Italy during the summer of 2020.

## Figures and Tables

**Table 1:** Number of total COVID-19 deaths, contagions and swab tests and of daily ICU and non-ICU patients during the summers of 2020 (20 June 2020–22 September 2020), 2021 (21 June 2021–22 September 2021) and 2022 (21 June 2022–23 September 2022).

Summer	2020	2021	2022
COVID-19 deaths	1085	3236	9044
COVID-19 contagions	62,540	392,322	4,283,835
COVID-19 swab tests	5,717,867	20,250,565	21,713,992
COVID-19 daily ICU	91	351	282
COVID-19 daily non-ICU	1198	2685	7192

## Data Availability

Publicly available datasets were analyzed in this study; further requests for data should be addressed to the corresponding author.
